# Trends of Foodborne Diseases in Mississippi: Association with Racial and Economic Disparities

**DOI:** 10.3390/diseases9040083

**Published:** 2021-11-13

**Authors:** Luma Akil

**Affiliations:** Department of Behavioral and Environmental Health, School of Public Health, Jackson State University, Jackson, MS 39217, USA; luma.akil@jsums.edu

**Keywords:** foodborne diseases, *Salmonella*, *Campylobacter*, *Shigella*, health disparities, socioeconomic status, Mississippi

## Abstract

Background: Foodborne diseases are a major source of concern in USA. These diseases are a burden on public health and significantly contribute to the cost of health care. There is an urgent need to understand the contributing factors for such outbreaks, especially in Mississippi (MS), an agricultural state with low socioeconomic status. Methods: Secondary data for the current study were obtained from the Mississippi State Department of Health (MSDH) Epidemiology department for the study period 2010–2018. Data were for individuals with reported foodborne diseases cases. The data were analyzed to determine the pathogens’ trend over time, the highest contributing pathogens to foodborne diseases, the significant geographical variation, and any significant differences in rates based on demographic variables. Results: *Salmonella* was the highest contributing pathogen to foodborne disease in MS. The study showed a seasonal variation in the trends of pathogens and a geographical variation, and no racial differences in the incidents of the foodborne diseases was observed. Conclusions: Incidence rates of foodborne illness remain high in the state of Mississippi. A better understanding of high levels of foodborne infections caused by *Salmonella*, *Shigella*, and *Campylobacter* resulting from cultural food handling practices or socioeconomic factors will allow to provide guidelines and food safety preventive measures.

## 1. Introduction

Foodborne diseases are an important and growing public health concern and contribute to a high morbidity and mortality in the United States (U.S.) and worldwide [1]. It was estimated that foodborne diseases contribute to about 9.4 million cases per year in the United States, resulting in 1351 deaths [1]. Over 90% of these diseases result from ingestion of contaminated foods or beverages with around 15 major pathogens, including viruses, bacteria, and parasites. The most common pathogens are *Norovirus* and *Salmonella* [2,3]. Common symptoms of foodborne diseases include nausea, vomiting, abdominal pain, diarrhea, lack of appetite, and fever. The severity of these symptoms depends on several factors, including the pathogen involved, infectious dose, health conditions of the infected individual, and others. Foodborne diseases also cause significant economic losses and contribute to about 15.6 billion dollars every year of economic loss in the U.S. [4].

In the state of Mississippi (MS), foodborne diseases continue to be a burden on public health. The Mississippi State Department of Health (MSDH) indicated that the most common foodborne diseases in Mississippi are salmonellosis, campylobacteriosis, and shigellosis [5]. Mississippi is one of the leading agriculture states with low socioeconomic status and high rates of obesity and associated health disparities. Poverty and low socioeconomic status are linked with under-detection of foodborne diseases cases because of availability of health insurance or financial means to seek healthcare [6,7].

Incidence rates of foodborne diseases have not traditionally been tracked by race, ethnicity, or income. A limited number of studies have found that low-income populations are more likely to experience greater rates of gastrointestinal illness and that individuals of minority racial and ethnic groups suffer from greater rates of some foodborne diseases [8].

The southern parts of the USA are more vulnerable to increase outbreaks of foodborne diseases due to socioeconomic status, climatic changes, and agricultural practices [5,6,7]. Our previous studies examined the effects of climatic changes on *Salmonella* infections in the southern states, and the results showed a significant effect of increased temperature on the rate of outbreaks and incidents of *Salmonella* [5]. Limited studies have examined foodborne diseases in the state of MS. It is critical to determine the extent of these diseases in the state and their correlation with socioeconomic status.

The objectives of this project are to (1) determine the major contributing pathogens to foodborne diseases in Mississippi from 2010–2018 and (2) determine the trends and differences in foodborne disease rates between geographically and socioeconomically different regions of Mississippi.

## 2. Materials and Methods

### 2.1. Data Collection

Foodborne disease incidents provide information about the pathogens and foods responsible for illness. Analyzing the trend of the foodborne disease cases will allow to identify the leading pathogens, the geographical area with higher cases, and the most vulnerable populations.

Data for the current study were obtained from the Mississippi State Department of Health Epidemiology department for the study period 2010–2018. Data for individuals with reported foodborne diseases were requested and included the following variables: pathogen and demographics, including race, age, gender, county, city, source of exposure, and other health conditions, if applicable. To further determine the racial differences in foodborne diseases, data of race percent and poverty rate for each county and district in Mississippi were collected from the United States Census Bureau. A correlational analysis was conducted between the race and foodborne diseases rates.

### 2.2. Statistical Analysis

The data were analyzed using SAS 9.4 (SAS institute, Cary, NC, USA) to determine the pathogens’ trend over time, the highest contributing pathogens to foodborne diseases, the pathogens responsible food for illness, the significant geographical variation, and any significant differences in rates based on demographic variables.

The prevalence rates of foodborne diseases were compared between race and gender using PROC GLM; in addition, PROC FREQ was used for the frequency analysis, and PROC ANOVA was used for Analysis of Variance to determine the significant differences in foodborne diseases’ incidents based on social and environmental factors. In addition, PROC REG/CORR was used to determine the correlation between poverty and race in addition to the rates of foodborne diseases.

## 3. Results

During the study period (2010–2018), 15,484 cases of foodborne diseases were reported by to the MSDH. The highest reported pathogens were *Salmonella* with about 80% of the cases, followed by *Campylobacter* with 15% and *Shigella* with about 10% of the total cases (Table 1).

Trend and Seasonality: Analysis of variance and time-series analysis were carried out to determine the pathogens rates’ change over time and the seasonality effect. For this analysis, only the highest contributing pathogens were included (*Salmonella*, *Campylobacter*, *Shigella*, and *E. coli*).

**Figure 1:**
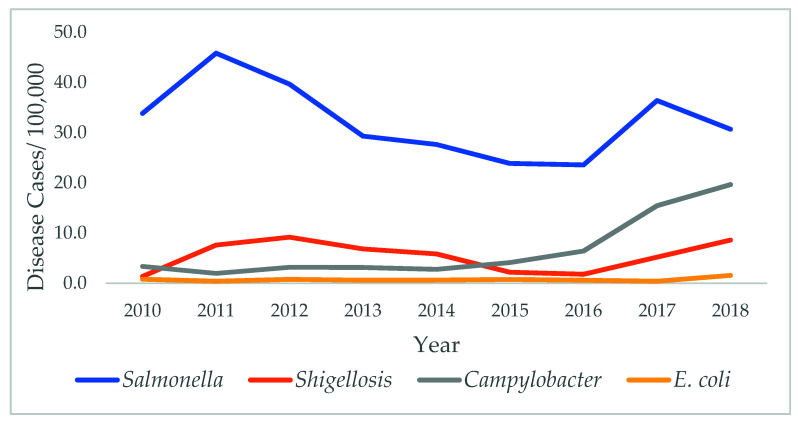
Time-series analysis for the pathogens between 2010 and 2018.

The number of *Salmonella* cases remains significantly higher compared with other foodborne diseases. A significant change over time was observed for *Salmonella* (*p* < 0.001): the highest rates were in 2011, with an annual average of 46 cases/100,000. A significant increase in the rates of shigellosis and camplybactorsosis was observed during the study period as well (*p* < 0.001), with the highest in 2018 (8.6/100,000 cases and 19.7/100,000 cases, respectively) (Figure 1).

**Figure 2:**
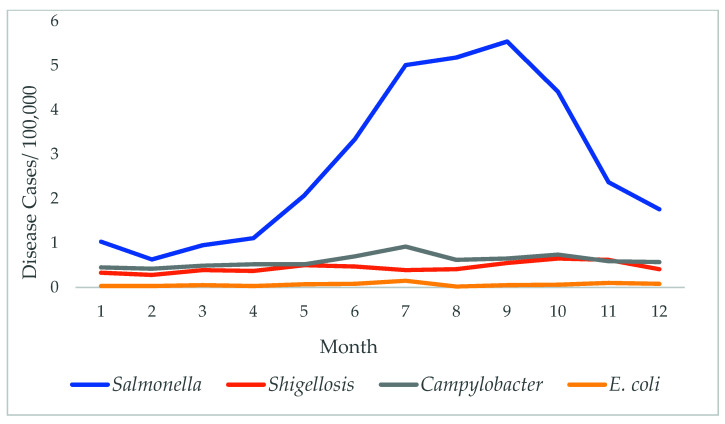
Seasonal trends of selected foodborne diseases. A seasonal trend was observed in *Salmonella* cases and *Campylobacter*.

A significant seasonal trend was observed for the *Salmonella* (*p* < 0.001), with the highest rates over the summer period, peaking in July through September. A similar trend was also observed with *Campylobacter* (*p* = 0.033), with the peak during July. No significant seasonality was observed for the other foodborne pathogens (Figure 2).

**Figure 3:**
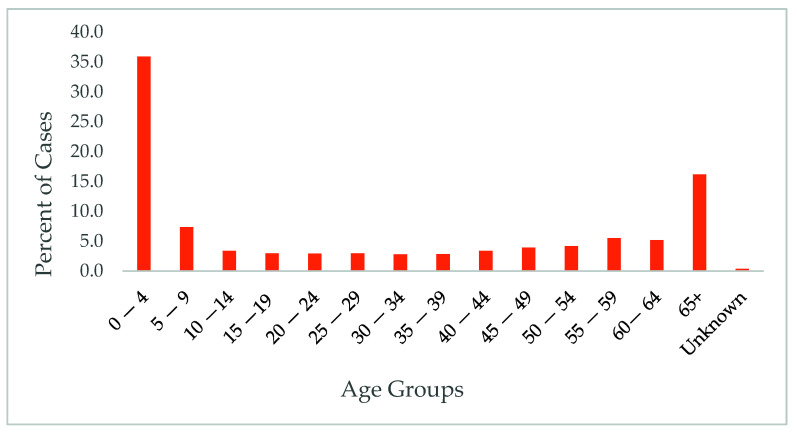
Age differences in foodborne diseases cases.

Age and gender differences: A significant difference was observed between the different age groups (*p* < 0.01), with the majority of reported cases affecting young children and the elderly (36% of the cases and 16%, respectively), as shown in Figure 3.

No significant difference in the incidence of foodborne diseases were observed between males and females (*p* > 0.05). Males showed a slightly higher rate than females (50.4% of the cases vs. 48.9%).

**Figure 4:**
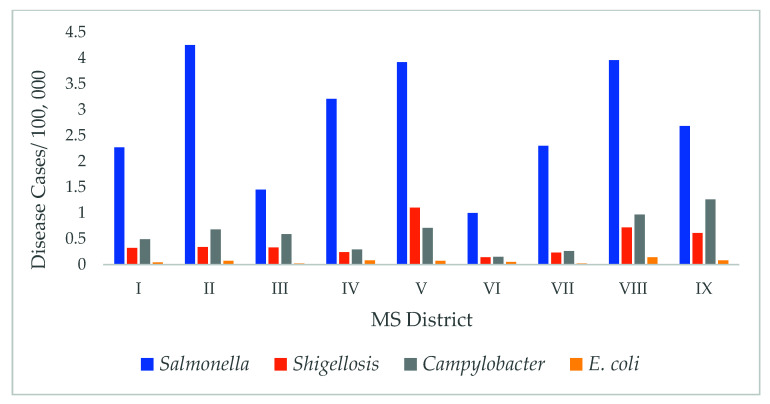
Geographical Variation between the foodborne diseases cases and the Mississippi districts.

Geographical differences: Significant regional variation was observed during the study period for all pathogens (*p* < 0.05). Highest rates of *Salmonella* were observed in regions 2 and 5 (northeast 4.2/100,000 average cases and west-central regions 3.9/100,000 average cases). The southwest region (VIII) showed the highest rates of *Campylobacter* and *E. coli* (0.9/100,000 and 0.13/100,000 average cases, respectively), while the coastal plains (IX) showed the highest rates of *Shigella* (0.6/10,000 average cases), as shown in Figure 4.

**Figure 5:**
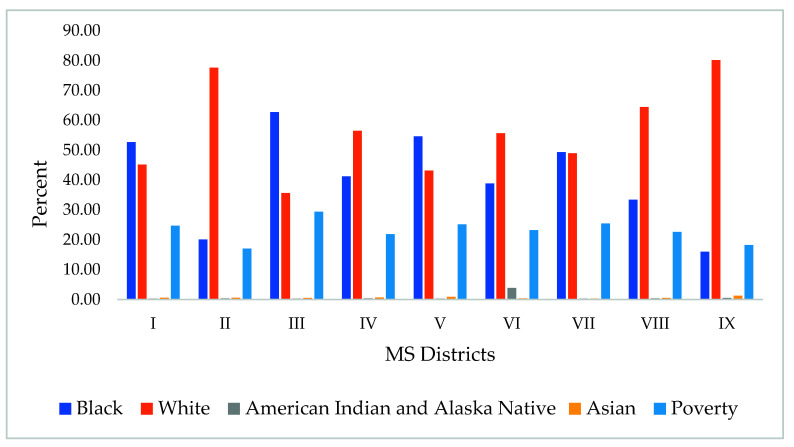
Geographical variation in poverty rate and the percent of racial populations and the Mississippi districts.

Racial differences: In this study, the majority of the foodborne disease cases were for white populations, accounting for 45% of the cases, while African Americans reported 14% of the cases. However, for more than 5000 cases (36%), race was not determined, as shown in Table 2. Race was significantly different between the different districts of the state (*p* < 0.05), as shown in Figure 5. The highest percent of Black population was observed in regions 3 and 5 (62% and 55%, respectively), while regions 9 and 2 had the highest White populations (80% and 77%, respectively). In addition, the poverty rate was also the highest in regions 3, 5, and 7. A significant correlation was observed between poverty and Black populations in MS districts (R^2^ = 0.896; *p* < 0.01). However, a weak negative correlation was observed between the rates of foodborne diseases and race and poverty. However, *Salmonella* rates were shown to be higher in regions with higher White populations, such as regions 8 and 4 (Table 3).

## 4. Discussion

*Salmonella* continues to contribute to the high rates of foodborne diseases in Mississippi, with an annual average of 32.3 cases/100,000 during the study period compared with 16.7/100,000 annual U.S. Average. The highest rates were in 2011 and 2017. Several *Salmonella* outbreaks occurring during both years were linked to beef, imported papayas, ground turkey, and nuts [9,10]; however, no specific outbreaks were linked with the significantly higher rates.

A significant increase over time in *Campylobacter* and *Shigella* rates was observed since 2015. According the CDC, several *Campylobacter* outbreaks were related to contact with puppies [11], while *Shigella* outbreaks were usually caused by eating contaminated food, swimming in recreational water, or from exposure to stool during sexual contact [12]. However, no significant changes were observed with *E. coli* incidents during the study period.

A seasonal trend was observed in *Salmonella* and *Campylobacter* infections. In our previous studies, we identified a strong positive correlation between high temperature and *Salmonella* incidents in MS. Our neural network models showed that an increase of 1 °F (0.55 °C) resulted in a four-case increase of *Salmonella* in MS [5]. Better growth of *Salmonella* at higher temperatures leads to higher concentration of *Salmonella* in the food supply in the warmer months. Inadequate cooking practices are also more common during these months (picnics, barbecues, etc.). Temperature may affect the transmission of *Salmonella* infections via several causal pathways, such as direct effects on bacterial proliferation and indirect effects on eating habits during hot days [5]. Studies have shown that diseases associated with climate change are estimated to comprise 4.6% of all environmental risks. In the year 2000, about 2.4% of diarrheal diseases and 0.3% of mortality in the world were contributed to climate change [13]. A similar finding from a study in Europe concluded that higher ambient temperatures have been associated with 5–10% higher salmonellosis notifications for each degree increase in weekly temperature for ambient temperatures above 5 °C. Roughly one-third of the transmission of salmonellosis in England and Wales, Poland, the Netherlands, the Czech Republic, Switzerland, and Spain can be attributed to temperature influences [13]. In African countries, foodborne diseases contribute to about 91 million illness and 137,000 death every year [14]. Extreme temperature and precipitation in the regions will impact enteric pathogens, particularly fecal-oral pathogens that are present in the environment, increasing the risk of gastrointestinal and diarrheal diseases that are responsible for 70% of the burden of these foodborne diseases [14]. Control measure and food safety guidelines will help to reduce such bacterial infections and limit the resulting consequences of climate change.

In the current study, foodborne diseases in MS were the highest among children younger than five years old. These findings are consistent with other studies, for example, a study observed that *Salmonella* Newport is higher among five-year-old and younger children [15]. Young children are more likely to be exposed to *Salmonella* through nonfoodborne routes, their exposure maybe resulting from crawling, outdoor play, and animal exposure [15]. In addition, mothers’ level of education was positively associated with food safety in children [16].

In addition, elderly populations also had higher rates of foodborne diseases and hospitalization. This is primarily caused by weak immune system and other health conditions that increase the risk of complications from such exposures.

No significant difference in the incidents of foodborne diseases was observed between males and females in this study. However, studies have suggested specific gender-related foodborne diseases based on the food sources. For example, males had higher cases of foodborne diseases associated with red meats, dairy, and shellfish, while females were more involved with outbreaks associated with vegetable raw crops, grains-beans, fruits, seeded vegetables, sprouts, and nuts-seeds. These studies demonstrates that the demographic distribution of outbreaks might result from the demographically related food preferences [17].

Substantial regional differences in the incidence of foodborne diseases in Mississippi were observed in this study. Rates of *Salmonella*, for example, were higher in highly populated regions of the state. In such areas, access to health care is more available and may contribute to a higher reporting of the cases. *Shigella*, on the other hand, was higher in the coastal plains area, where more swimming in recreational areas and consumption of undercooked seafood is high and can lead to such higher cases.

In our previous studies, we observed a significant variation in *Salmonella* and *E. coli* incidents among the MS districts using GIS mapping [6]. We determined a significant association between *Salmonella* infections and access to health care, poverty, and other socioeconomic variables [6]. Socioeconomic and demographic indicators can be used to predict which individuals and communities are at an increased risk of acquiring infections. A geographical variation was observed in other studies as well, where increases in incidence occurred at sites in the southern USA, and a northeastern state had the highest mean annual incidence in FoodNet [18].

Generally, socioeconomic status (SES) is an important predictor of several poor health outcomes, including chronic diseases, mental illnesses, and mortality. Studies have found that residents of low socioeconomic status (SES) areas and particularly areas with higher percent African American populations have greater access to smaller, independently operated food markets and fast-food/take-out restaurants compared to those of high SES. This differential access may result in an increased food safety risk for low-income and minority populations [8].

In this study, the highest rates of foodborne diseases were among the Caucasian/White population, with 45% of the cases, while only 14% of the cases were among the African American/ Black population. However, the race data were missing for about 36% of the cases. The racial composition of Mississippi is as follows: White: 58.41% and Black or African American: 37.72%, according to the most recent American Community Survey ACS [19]. The further analysis of census data based on the districts of the states showed that the racial composition of the state varied significantly in MS. In addition, a significant positive correlation was observed between poverty and Black populations in different districts. Even though a weak correlation was observed between foodborne diseases and race, salmonellosis was the only disease that showed higher incidents in districts with higher White populations. A very weak or negative correlation was observed between the different foodborne diseases and race and poverty rates in this study. These results indicate that access to health care is most likely to be the contributing factor for reporting foodborne diseases.

Other studies indicated that *Salmonella* incidence increased with higher education and income levels [20]. It was suggested that populations living in higher SES areas might have increased access to care and might be more likely to submit specimens regardless of disease severity, whereas populations in lower SES areas might seek care or diagnostic testing only when illness is serious or prolonged [20,21,22,23]. In addition, populations with higher SES usually are at high risk due to activities and behaviors such as international travel, consumption of high-risk food items (e.g., raw fruits and vegetables and undercooked meat), and eating at restaurants [8]. Food consumption at restaurants is more common among higher SES populations. Restaurants’ foodborne outbreaks are shown to be a major contributor to foodborne diseases, and this may result from food handling and preparation practices in restaurants, including inadequate thawing, resulting in pathogen proliferation and cross-contamination [21].

Social determinants of health, such as poverty, unemployment, and low education, are often correlated with race and ethnicity. Racial and ethnic minorities may receive a different level of care compared with non-minority patients; the non-Hispanic Black population consistently has poor access to healthcare in addition to having the highest rate of preventable hospitalization compared with other racial/ethnic groups in the United States [18]. It was also found that the Black population, particularly men 18–49 years old, experienced higher incidence of severe shigellosis, and it may be associated with disparities in social determinants of health, sexual orientation or behaviors, antimicrobial resistance, comorbidities, or circulation of more pathogenic strains within certain populations [18].

Further, a study that analyzed FoodNet data from 1998 to 2000 indicated that the incidence of *Salmonella enteric* serovar Enteritidis infection was highest among African Americans, followed by Hispanics and then Caucasians. It showed that minority populations suffer from a greater incidence of salmonellosis than Caucasians [8]. Further, studies also found a positive association between incidence of salmonellosis and the percent African American and Hispanic population [23].

Incidence rates of foodborne illness remain high in the state of Mississippi and need further investigation and identifying of the causes of such high incidents. A better understanding of high levels of foodborne infections caused by *Salmonella*, *Shigella*, and *Campylobacter* resulting from cultural food-handling practices or socioeconomic factors will allow to provide guidelines and food safety preventive measures.

## 5. Conclusions

In this study, a comprehensive analysis of foodborne diseases that are most common in the state of Mississippi was performed, and factors that may lead to such outbreaks were investigated. *Salmonella* remains the highest contributing pathogen foodborne diseases in MS compared with others. Several environmental and socioeconomic factors may contribute to such outbreaks. These include climatic/seasonal variability, such as increase in temperature; geographical variation between areas with different poverty, racial, and economic status; access and availability of health care; and gender and age variability. Further studies are still needed to understand these factors and to determine control and food safety guidelines. Foodborne diseases are major health concerns worldwide. The absence of reliable data on the burden of foodborne disease, especially among poor communities, obstructs understanding about its public health importance and prevents the development of risk-based solutions to its management.

## 6. Limitations

This project is limited by the availability and accuracy of the data. This study included data in the state of Mississippi for the period of 2010 through 2018 only. Several variables in the dataset were missing, which may lead to uncertainties in the interpretation of the results. In addition, data from multiple sources were used in this study, which may add to the limitation of the study. More research is needed and is critical in disadvantaged states, such as Mississippi.

## Figures and Tables

**Table 1:** Percent of reported pathogens during the study period 2010–2018.

Pathogen	Percent
*Campylobacter*	**15.5**
*Escherichia coli* O157:H7	0.9
*Hepatitis A*	0.4
Listeriosis	0.4
Non-O1 And Other Vibrio spp.	0.4
Salmonellosis	**80.1**
Shiga Toxin-Producing *E. coli* (Stec)	**1.6**
Shigellosis	**9.35**
*Vibrio parahaemolyticus*	0.2
*Vibrio vulnificus*	0.4

**Table 2:** Frequency of foodborne diseases by race and ethnicity.

Race	Percent
American Indian or Alaska Native	0.3
Asian	0.3
**Black or African American**	**14.1**
Black or African American; Other Race	0
Black or African American; White	0
Native Hawaiian or Other Pacific Islander	0
Native Hawaiian or Other Pacific Islander; Asian	0
Other Race	4.1
Other Race; Black or African American	0
Other Race; White	0
**Unknown**	**36**
**White**	**45.1**
White; Asian	0
White; Black or African American	0
White; Other Race	0
**Total**	**100**

**Table 3:** Rates of racial composition (%), poverty (%), and foodborne diseases (monthly average/100,000) in different districts of MS.

District	Black	White	American Indian And Alaskan Native	Asian	Poverty	*Salmonella*	*Shigella*	*Campylobacter*	*E. coli*
I	52.70	45.20	0.31	0.60	24.69	2.27	0.32	0.49	0.04
II	20.07	77.62	0.37	0.60	17.07	4.25	0.34	0.68	0.07
III	62.73	35.63	0.29	0.50	29.38	1.45	0.33	0.59	0.02
IV	41.28	56.52	0.40	0.65	21.88	3.21	0.24	0.29	0.08
V	54.61	43.20	0.29	0.93	25.14	3.92	1.10	0.71	0.07
VI	38.89	55.67	3.88	0.39	23.27	1.00	0.14	0.15	0.05
VII	49.32	49.01	0.33	0.32	25.48	2.30	0.23	0.26	0.02
VIII	33.41	64.50	0.38	0.53	22.62	3.96	0.72	0.97	0.14
IX	16.00	80.20	0.57	1.28	18.28	2.68	0.61	1.26	0.08

## Data Availability

Data are available by request form the Mississippi state Department of Health.
